# High speed e-beam writing for large area photonic nanostructures — a choice of parameters

**DOI:** 10.1038/srep32945

**Published:** 2016-09-16

**Authors:** Kezheng Li, Juntao Li, Christopher Reardon, Christian S. Schuster, Yue Wang, Graham J. Triggs, Niklas Damnik, Jana Müenchenberger, Xuehua Wang, Emiliano R. Martins, Thomas F. Krauss

**Affiliations:** 1 State Key Laboratory of Optoelectronic Materials and Technologies, School of Physics, Sun-Yat Sen University, Guangzhou, 510275, China; 2 Department of Physics, University of York, York, YO10 5DD, UK; 3 Raith Service & Support Team, Raith GmbH, 44263, Germany; 4 School of Engineering of São Carlos, University of São Paulo, Av. Trabalhador Sãocarlense, 400, São Carlos-SP, Brazil

## Abstract

Photonic nanostructures are used for many optical systems and applications. However, some high-end applications require the use of electron-beam lithography (EBL) to generate such nanostructures. An important technological bottleneck is the exposure time of the EBL systems, which can exceed 24 hours per 1 cm^2^. Here, we have developed a method based on a target function to systematically increase the writing speed of EBL. As an example, we use as the target function the fidelity of the Fourier Transform spectra of nanostructures that are designed for thin film light trapping applications, and optimize the full parameter space of the lithography process. Finally, we are able to reduce the exposure time by a factor of 5.5 without loss of photonic performance. We show that the performances of the fastest written structures are identical to the original ones within experimental error. As the target function can be varied according to different purposes, the method is also applicable to guided mode resonant grating and many other areas. These findings contribute to the advancement of EBL and point towards making the technology more attractive for commercial applications.

Photonic nanostructures have become ubiquitous in modern society. They appear on credit cards as security features^1^,^2^,^3^, are used for the coupling of light in LEDs^4^,^5^,^6^,^7^ and solar cells^8^,^9^,^10^,^11^ and provide added functionality in optoelectronic applications^12^,^13^,^14^. They can be generated by interference lithography^15^, nanoimprint lithography^16^,^17^ and related techniques. Some high-end applications, however, as well as the masters used for nanoimprint, require the pattern to be directly written by electron-beam lithography (EBL). Since EBL is generally a serial process, the associated costs scale directly with pattern size and writing speed^18^,^19^. The writing speed can be improved by increasing the beam current, but higher beam currents result in larger spot sizes due to the intrinsic repulsion of electrons. The question is therefore to what extent the beam current can be increased without loss of pattern fidelity and without loss of photonic performance. To answer this question, we introduce a target function as a guideline for designing the patterns and evaluating the results. The target function reflects the functionalities that the nanostructures should exhibit, while not focusing on the structures themselves. As an example, for applications in solar cells, the purpose of these nanostructures is to enhance light harvesting in thin absorbing films. Based on this aim, we designed nanostructures whereby the fidelity of the Fourier Transform spectrum is applied as a target function to optimize the writing speed of EBL. The choice of the Fourier Transform as the target function is motivated by the strong dependence of light trapping performance on the Fourier properties of the coupling surface^20^. In particular, we have varied the beam current and a number of other parameters, such as area step size, development time and pattern generation method.

The nanostructures we selected are known as quasi-random supercells^20^. The structures appear random at the level of the unit cell, yet the unit cell is repeated periodically. These nanostructures have previously been shown to provide some of the most effective light trapping performance for thin film solar cells^20^,^21^. The operational principle of the quasi-random supercell is to enhance the higher diffractive orders of their Fourier spectra, whilst suppressing the lower orders^22^. These Fourier properties enable broadband coupling of quasi-guided modes into thin film materials. Since the broad band absorption is predominantly determined by the Fourier properties, structures that have similar Fourier characteristics to the optimum case will exhibit similar high performance^23^. For example, Fig. 1 shows different patterns with similar Fourier spectra, which result in similar absorption properties, shown in Fig. 1f (calculated absorption spectrum). Indeed, from the numerical simulation, the integrated absorption spectrum varies only within ±2% between these patterns.

In this paper, we verify the fidelity of the Fourier Transform spectra as the target function against parameters that influence the EBL writing speed. Additionally, and more importantly, we achieve a 5.5-fold reduction of the lithographic exposure time without loss of photonic performance.

## Results and Discussion

The total processing time 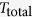 used for EBL can be separated into exposure time 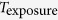 and settling time 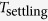 (equation (1)). While the exposure time, as the first term in equation (1), is mainly determined by the writing frequency *F* and number of pixels 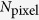 (equation (2)), the settling time is related to the pattern complexity and so directly linked to the software processing procedure. For most e-beam machines, 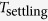 is set by the write field and the overhead of the pattern generator and beam blanker^18^. However, since we fixed the write field to a size of 500 μm × 500 μm, the settling time here is dominated by the pattern generator and beam blanker. The writing frequency *F* then drives the actual writing speed (the frequency with which the beam is stepped from one pixel to another). For our pattern generator, the highest frequency *F* is 50 MHz. Within these boundaries, the exposure time for a single pixel 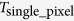 is inversely proportional to the frequency and is determined by three factors: dose, current, and step size (SS) (equation (3)). In the following section, we discuss the influence of each factor on the exposure time, and characterize the corresponding optical properties.


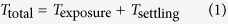



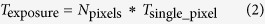



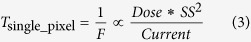


where 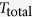 is the time for whole processing, 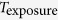 is the time for pure e-beam writing, 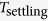 is the time for settling parameters and include overhead time, 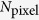 is the number of pixels that a pattern is divided into by pattern generator, 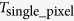 is the time required to expose a single pixel, 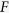 is the machine’s writing frequency (in 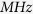), 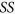 is the step size (in μm) and 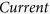 stands for beam current (in 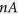).

## Beam Current

**Figure 2:**
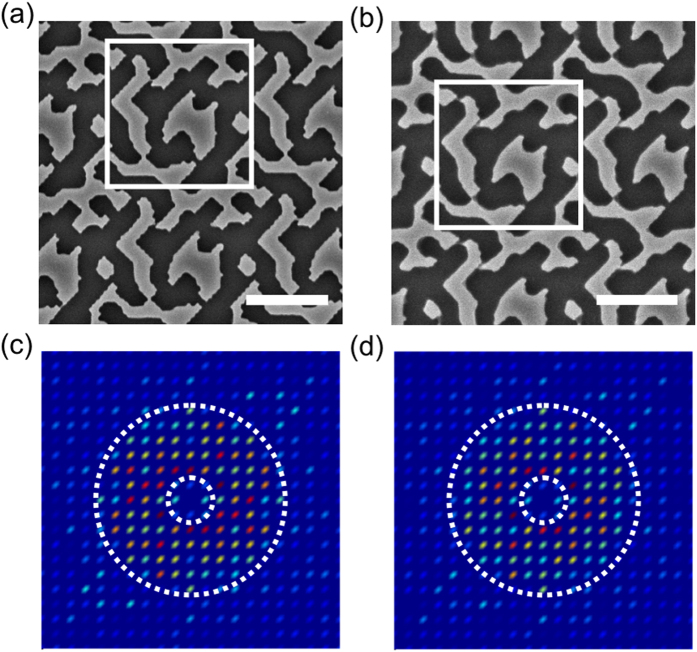
Comparison of 32 bit quasi-random patterns made using different current modes. (**a**) Pattern written at a current of 0.61 nA. (**b**) Pattern written at a current of 4.86 nA. (**c**) The corresponding Fourier transform spectrum of (**a**). (**d**) The corresponding Fourier transform spectrum of (**b**). The unit cell is represented by the white rectangle. The scale bar is 1 µm.

The spot size of the electron beam is influenced by the beam current via the aperture, and is one of the most important parameters to define writing speed and resolution. Typically, one obtains a higher resolution by choosing a lower current (e.g. 0.61 nA, Beam diameter ~5 nm^24^), while the speed is improved by applying a larger current (e.g 4.86 nA, Beam diameter ~10 nm^24^). A comparison of the same pattern written with different beam currents is shown in Fig. 2 and Table 1. The patterns (Fig. 2a,b) look different, but will the light trapping performance be affected?

In order to quantify the light trapping performance, we compare the Fourier spectra (Fig. 2c,d). Both spectra are very similar and preserve the desired ring shape. In order to verify whether the optical properties are affected, we then compare the light harvesting performance by conducting a 3D FDTD simulation. The SEM image was first converted into a black and white binary picture. This binary picture was then used to build the 3D model, which closely resembles the real pattern. In this way, the pattern distortion is included in the model. In the model, the material is set to be c-Si, the film thickness is 400 nm, the etch depth 100 nm, the backside of the structure is attached to a metal as a perfect conductor, the radiation is incident perpendicularly and the light source is un-polarized. The simulation then yields the absorption spectrum, which we use to calculate the short-circuit current 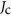 by integration over the standard solar spectrum AM 1.5G^25^:


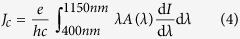


where *e* is the elementary charge, *h* the Planck constant, c the speed of light in vacuum, λ the wavelength, A(λ) the absorption, d*I*/d*λ* the Solar Spectrum of AM 1.5 G. Notice that this calculation of 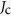 (equation (4)) does not consider electrical losses, however it is useful for comparing the optical properties of the different structures.

The result is that the short-circuit currents for the two writing modes are both 19.00 mA/cm^2^, highlighting that different patterns can yield the same performance. Since the light trapping performance is governed by the Fourier spectrum^20^, our assumption is confirmed that the Fourier spectrum and not the actual pattern shape is the determining feature of the nanostructure. Further information on how the spatial resolution affects the Fourier spectra can be found in the Supplementary Material. In terms of writing time, the high current mode is significantly superior, and we achieve an improvement in reducing the exposure time from 24 to 9.5 minutes for a 1 mm^2^ area pattern.

## Step Size

The step size is the distance between the steps of the beam as it writes the pattern. As shown in equation (2), the total exposure time is the product of the number of pixels and the time per pixel. When increasing the step size, we reduce the number of pixels to be written and therefore the number of beam movements and the corresponding overhead (beam settling time and etc.). As the step size increases, however, the pattern also becomes coarser, so we need to establish the impact of the beam overhead and understand the balance between the step size and the pattern distortion.

**Figure 3:**
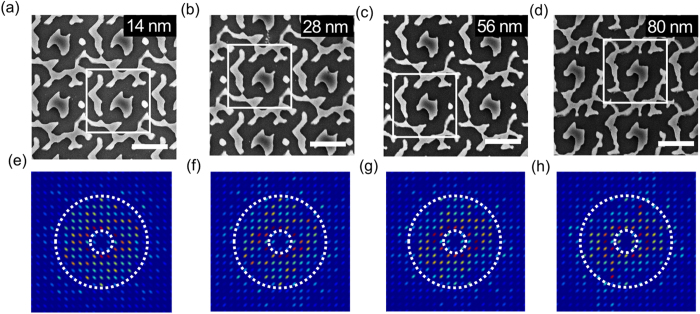
Comparison of patterns written with different area step sizes. (**a**–**d**) represent step sizes between 14 nm and 80 nm, (**e–h**) are the corresponding Fourier transform spectra. The scale bar is 1 µm.

Figure 3 shows patterns written with different area step sizes. From Fig. 3a–d, the shape of the pattern noticeably changes with increasing area step size. We note that even though a larger step size causes a loss of pattern quality and reduces the number of Fourier components, as apparent from Fig. 3a–h, we still get a relatively high short-circuit current compared to the bare silicon thin film with equivalent volume (thickness ~350 nm), as shown in Table 2. By increasing the step size from 14 nm to 80 nm, we get a reduction of exposure time from 9.5 to 7.5 minutes for a pattern of 1 mm^2^. This exposure time reduction can be understood as the reduction of dose and overhead time. Because each step size carries a tiny overhead in terms of beam settling time, and these overheads add up to be significant enough to be measured, even if the total dose is identical. As our e-beam system employs electro-static deflection, therefore, the beam movements are extremely fast. However, older, less expensive systems tend to employ electro-magnetic deflection and hence require beam settling times which makes them slower. Since the beam overhead is obviously low and the impact of the area step size on performance is larger than, for example, the beam current, we decided to use an area step size of 14 nm for the remaining experiments.

## Dose and Development Conditions

The dose to clear depends not only on the electron energy (low-energy electrons expose the resist with lower dose), the resist and substrate (backscattered electrons), but also on the development conditions (a function of developing time and temperature). Hence, we next consider the development time and temperature as a parameter.

**Figure 4:**
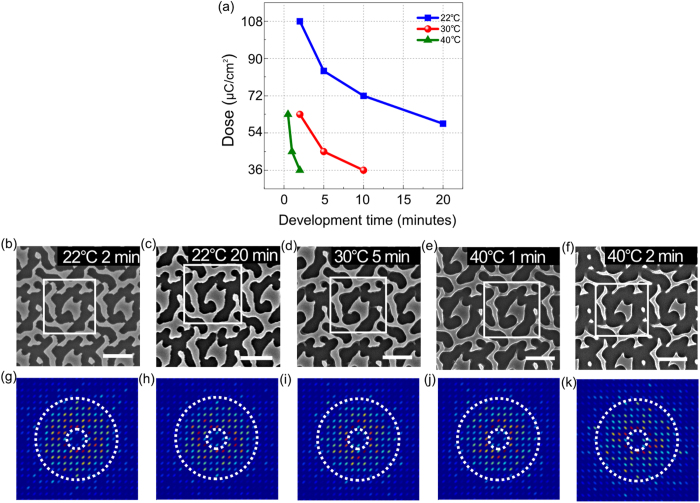
Dependence on development condition. (**a**) Relationship of dose, development time and temperature. (**b**–**f**) SEM micrographs of patterns developed at different times and temperatures. We note that (**b–e**) look very similar, while distortions appear in (**f**), which are also confirmed by the reduced short-circuit current (Table 3). (**g–k**) The corresponding Fourier transform spectra. Note scale bars in SEM images are 1 µm.

When using a development temperature of 22 °C, 2 minutes is required for full development of the pattern at a dose of 108 μC/cm^2^. When the development time is extended to 20 minutes, however, we note that a lower dose is needed. A known limit for minimum dose in the resist is the electron shot noise. Shot noise is usually probed as minimum dose for a given line-edge roughness and resolution. It is noted that by considering the feature uniformity, the lower dose we achieved here may not be the limit for minimum dose of the resist. Similarly, for higher temperatures, the development time for a dose of 45 μC/cm^2^ is reduced to 5 minutes at 30 °C or 1 minute at 40 °C, as shown in Fig. 4a and Table 3. Considering the balance of pattern fidelity, photonic performance and writing speed, we chose a dose of 58.5 μC/cm^2^ as our target value, i.e., the conditions used in Fig. 4c. We note that even when the pattern is exposed at lower dose, the Fourier spectrum still retains its ring shape within the targeted region. Commensurately, the absorption spectrum and the short-circuit current remain almost constant, as shown in Table 3. Therefore, increasing the development time and temperature allows us to reduce the exposure dose and the writing time without significant loss of pattern quality. We note, however, that the improvement in exposure time is only 10–20%, while the exposure dose is reduced by a factor of 2. This suggests that 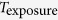 in equation (1) is close to its limit and that we need to consider 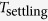 next.

## Settling Overhead

The settling overhead associated with writing the pattern consists of translating the pattern from a graphics format to machine code on-the-fly. For simple patterns, this is not an issue, as the computer is always faster than the beam, and the method is very efficient. For the rather complicated pattern consisting of many small elements used here, however, the computational translation presents an overhead that cannot be ignored. Therefore, we converted the content of a single write-field into a binary file before writing and then stepped this file across the sample. As a result, we were able to decrease the computation time by a factor of 1.9 (at 4.86 nA).

## Comparision and Discussion

Table 4 summarizes the different steps taken here and shows which parameters can be used to decrease the e-beam exposure time. For the data shown in the Table 4, the area step size was set to 14 nm and the development temperature to 22 °C. We include data for 136 pA for comparison, as this beam current level is relevant for many EBL tools based on converted SEMs.

**Figure 5:**
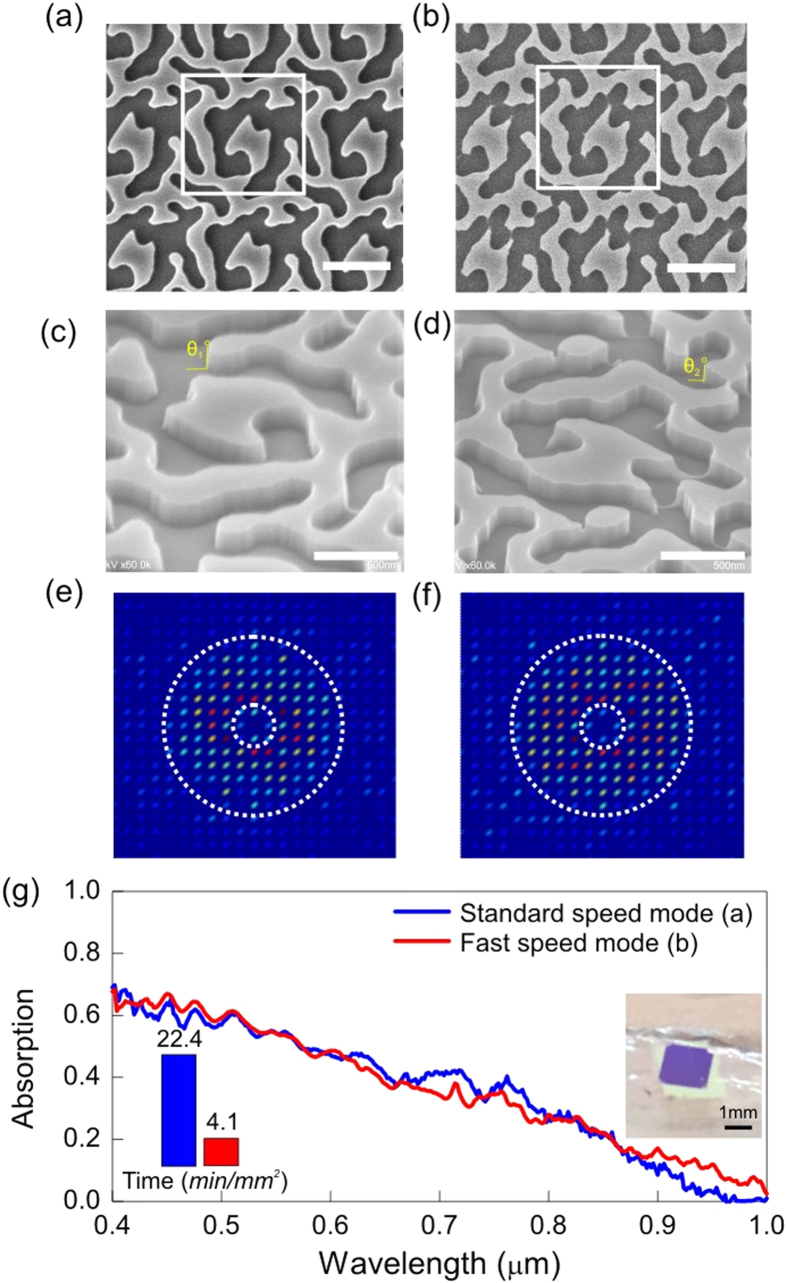
Comparison between standard speed mode and fast speed mode for quasi-random structures. (**a**) SEM of standard speed mode with writing current of 0.59 nA. (**b**) SEM of fast speed mode with writing current of 4.44 nA. (**c**,**d**) corresponding SEM viewed at 45°, showing the slope of sidewall. (**e**,**f**) Corresponding Fourier spectrum. (**g**) Measured absorption spectra. The silicon around the pattern was etched away. The short-circuit current calculated from measurement for standard speed mode and fast speed mode are 13.7 mA/cm^2^ and 13.2 mA/cm^2^, respectively. Insert: Left bottom, exposure time for standard mode (blue) and fast speed mode (red), right middle, a sample fabricated on glass. Scalar bars in (**a**) and (**b**) are 1 μm, in (**c**,**d**) are 0.5 μm.

In order to confirm the simulation result of short-circuit current and support the overall conclusion, we fabricated a 32 bit quasi-random pattern on a C-Si film on glass coverslip and measured the absorption spectra. Note that for this experiment, the silicon film is 500 nm thick, and there is no metal reflector covering the back side of the structure. Hence the absorption spectra and the implied short-circuit currents are different from those obtained in the previous sections; nevertheless, they serve as a good experimental comparison. Figure 5 compares the results of the sample prepared with the starting conditions and that prepared with the final conditions. Impressively, the absorption performance is almost equal while the exposure time is reduced from 22.4 minutes to 4.1 minutes for a 1 mm^2^ writing area, the writing speed is improved by a factor of 5.5. Furthermore, we checked the slope of the sidewall of the etched structures in order to investigate whether the writing speed impacts on the resist profile. As shown in Fig. 5c,d, the sidewalls of the final structure preserve their verticality after dry etching, which implies that the method we developed here has no apparent influence on the slope of the sidewall.

**Figure 6:**
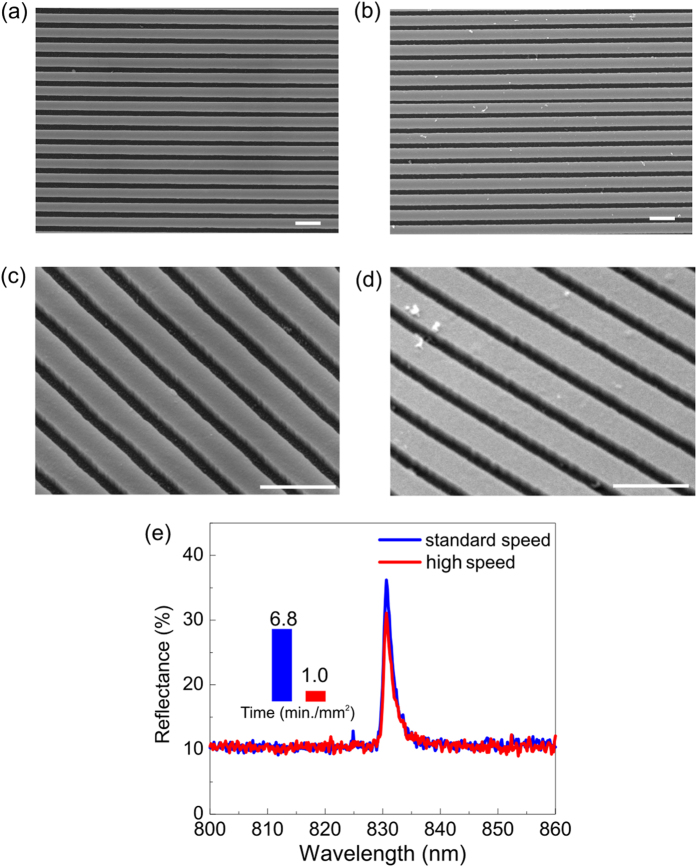
Comparison between standard speed mode and high speed mode for fabricating guided mode resonant gratings. (**a**) SEM of standard speed mode with beam current of 0.855 nA. (**b**) SEM of fast speed mode with beam current of 4.001 nA. (**c**,**d**) corresponding SEM viewed at 30 degree. (**e**) Measured resonance in TM polarization. The Q-factor from measurement of standard speed mode and high speed mode are 499 and 486. Insert: the exposure time for standard speed and high speed mode are 6.8 and 1.0 min./mm^2^. The scale bars in all SEM micrographs are 1 μm long.

As a further demonstration for the general applicability of our method, we used a guided mode resonance to demonstrate that improved writing speed is no impediment to high quality photonic functionality. Guided mode resonances^26^ are prime candidates for novel biosensors and resonant imaging modalities^27^. Here, we use the Q factor of the resonance as the target function to reduce the exposure time for fabricating resonant grating. The grating used here is fabricated in 150 nm thick Si_3_N_4_ film on glass, the period of the grating is 560 nm and the fill factor is 80% (Fig. 6). We compare two gratings, the first exposed with an aperture of 70 μm, a beam current of 854.9 pA and is developed for 2 minutes (Fig. 6a,c). The second grating is exposed with an aperture of 100 μm and a beam current of 4.0 nA and is developed for 20 minutes (Fig. 6b,d). Correspondingly, the exposure time for a 1 mm^2^ grating is reduced by a factor 6.8, from 6.8 minutes to 1 minutes, while the Q of both gratings remained almost the same (499 v.s. 486), as shown in Table 5. Therefore, the advantage in writing speed, as in the previous light trapping case, does not carry a penalty in terms of device functionality.

## Conclusion

Motivated by the need to reduce the exposure time of EBL for large area photonic nanostructures, we use a target function to optimize the process. We find that increasing the beam current and pre-processing the data has major benefits on the exposure side, especially for the type of complex discontinuous patterns used here, while increasing the development time and developer temperature allows for a decrease in exposure dose on the resist side. Overall, we have been able to reduce the writing time by a factor of 5.5 without compromising the light trapping performance of the device. Furthermore, we demonstrate that this method is also applicable to other photonic structures with a different functionality; by comparing two resonant gratings fabricated with high resolution, slow speed mode and another in high speed mode, we have demonstrated that a speed improvement by factor 6.8 does not impact on the quality factor of the resonance obtained. Therefore, we believe that our findings are transferable to many other applications of EBL and trust that they will help to make the technique more cost-competitive.

## Methods

### Quasi-random structure design

The quasi-random structure was designed by a direct binary search algorithm. Details can be found in reference^20^.

### Fourier transform spectrum

The Fourier Spectra were obtained from SEM images. First, the SEM images were converted into binary data, then the Fast Fourier Transform (FFT) method was employed to obtain the 2D Fourier Spectra.

### Silicon film fabrication

A 500 μm thick silicon wafer (DB Technologies Ltd.) was used for the e-beam writing tests, while we used a 500 nm thick c-Si film transferred onto a 1 mm thick glass slide for the optical measurements. The sample was spin-coated with 300 nm of positive electron sensitive resist AR-P 6200.09 (ALLRESIST GmbH) and then baked at 180  °C for 5 minutes. We chose a pattern consisting of a 32 bit quasi-random supercell with a minimal single pixel size of 56 nm^20^, which is optimized for diffractive light trapping and is ideal for this e-beam fabrication comparison. The pattern was defined by e-beam exposure on a Raith Voyager system operating at 50 kV followed by development in Xylene at varying temperatures. The patterns for measurement were also transferred from resist to Si film by dry etching with a mixture gas of CHF_3_ and SF_6_ (14.5:12.5) for 1 minute.

### Absorption measurement

The measurement setup^28^ consists of a white light source (LEUKOS SM 30), monochromator (Zolix, Omni λ 1509), PIN femtowatt silicon detector (Thorlabs, PDF10A), digital multimeter (Keithley, 2110 DMM) and a standard integrating sphere. The sample was placed in the middle of the integrating sphere. The absorption (*A*) of the final structure is calculated from *A* = 1 − *R* – *T*, where *R* is the total reflectance and *T* the total transmission.

### Errors and limitation

We note that there are errors associated both with the simulation and the experimental measurements. We find that the simulation yields slightly different values for the short circuit current depending on the choice of unit cell, and that the experiment has a measurement errors associated with it, which in both cases amounts to ±5%. And this throughput optimization guided by photonic performance approach is clearly limited to photonic designs that do not rely on extra fine features. In addition, even though the quoted lithography parameters are relevant for specific typologies of e-beam systems, the methods presented here are generic.

## Additional Information

**How to cite this article**: Li, K. *et al*. High speed e-beam writing for large area photonic nanostructures — a choice of parameters. *Sci. Rep.* **6**, 32945; doi: 10.1038/srep32945 (2016).

## Supplementary Material

Supplementary Information

## Figures and Tables

**Figure 1:**
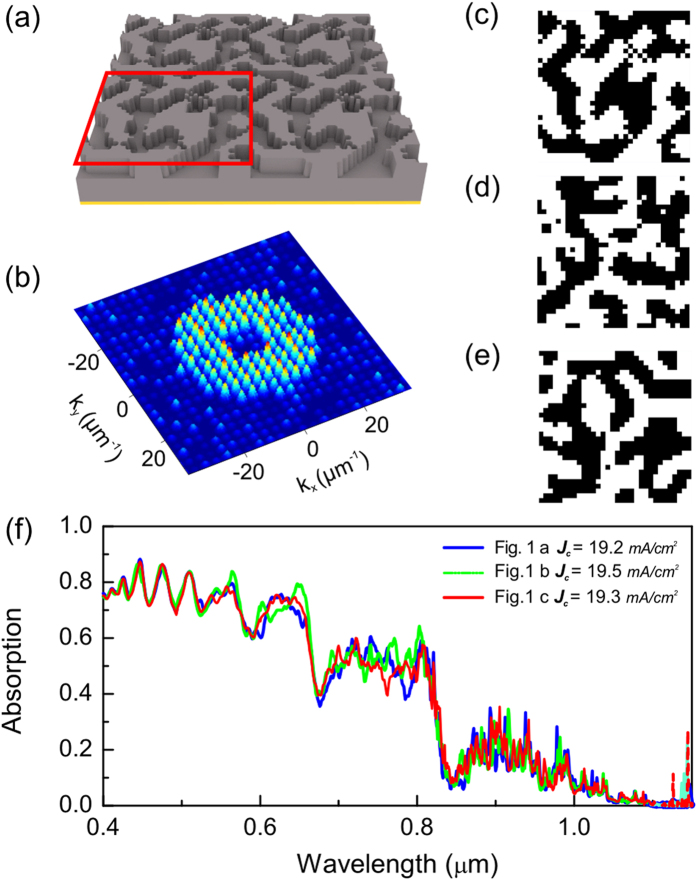
Quasi-random supercell and Fourier transform spectra. (**a**) Example of a quasi-random supercell. The red framed region is the unit cell, designed for light harvesting with (**b**) a ring shaped Fourier transform spectrum, which enhances the 2^nd^ to 6^th^ diffractive orders. The period of all supercells is 1.792 μm. (**c–e**) patterns which have similar Fourier transform spectra. (**f**) Corresponding absorption spectra for a 0.4 μm thick silicon thin film assuming a 100% reflective mirror layer at the back side. The calculated short-circuit current refers to the global solar spectrum AM 1.5G^25^ and assumes 100% internal quantum efficiency. Even though the 3 patterns look rather different in real space, their Fourier distributions and absorption spectra are very similar.

**Table 1:** Comparison of different current.

Current (nA)	Dose (μC/cm^2^)	Writing time for 1 mm^2^ (min.)	Short circuit current (mA/cm^2^)
0.61	108	24	19.0
4.86	108	9.5	19.0

(Step size is 14 nm, and development time is 2 minutes at 22 °C).

**Table 2:** Comparison of different area step size.

Quasi-random structure			C-Si slab
Area step size (nm)	Writing time for 1 mm^2^ (min.)	Short circuit current (mA/cm^2^)	Short circuit current (mA/cm^2^)
14	9.5	19.0	6.1
28	9.0	18.1	
56	8.5	17.5	
80	7.5	17.4	

(All patterns were written at a current of 4.86 nA and developed at 22 °C for 2 minutes).

**Table 3:** Development time v.s. temperature.

Temperature (°C)	Development time (min.)	Dose (μC/cm^2^)	Writing time for 1 mm^2^ (min.)	Short circuit current (mA/cm^2^)
22	2	108	9.5	19.0
22	20	58.5	8.9	18.3
30	5	45	8.4	18.1
40	1	45	8.4	18.1
40	2	36	8.2	17.1

(All patterns were written at a current of 4.86 nA).

**Table 4:** Comparison of different steps.

Current (nA)	Development time (min.)	Dose (μC/cm^2^)	preprocessing of data file	Writing time for 1 mm^2^ (min.)	Short circuit current (mA/cm^2^)
0.136	—	108	No	89.6	—
0.136	—	58.5	No	49.4	—
0.136	—	58.5	Yes	46.6	—
0.63	2	108	No	23.7	19.0
0.63	20	58.5	No	16.0	18.7
0.63	20	58.5	Yes	11.9	18.2
4.86	2	108	No	9.5	19.0
4.86	20	58.5	No	8.9	18.3
4.86	20	58.5	Yes	4.7	18.0

(Development temperature is at 22 °C).

**Table 5:** Comparison of the different writing conditions for guided mode resonances, and corresponding grating performance.

Current (nA)	Development time (min.)	Dose (μC/cm^2^)	Writing time for 1 mm^2^ (min.)	Resonance (TM)	
				FWHM (nm)	*Q*
0.855	2	145	6.8	1.66	499
4.001	20	70	1.0	1.71	486

(Area step size are set to 16 nm).
