# Supplementary material for: High speed e-beam writing for large area photonic nanostructures — a choice of parameters
Source: Sci Rep. 2016 Sep 16;6:32945. doi: 10.1038/srep32945 (PMC5025733; doi:10.1038/srep32945)
Supplement: Supplementary Information [file srep32945-s1.doc]

**SUPPLEMENTARY INFORMATION**

High speed e-beam writing for large area photonic nanostructures – a choice of parameters

Kezheng Li^1, 2, †^, Juntao Li^1, †^, Christopher Reardon^2^, Christian S. Schuster^2^, Yue Wang^2^, Graham J. Triggs^2^, Niklas Damnik^3^, Jana Müenchenberger^3^, Xuehua Wang^1^, Emiliano R. Martins^4^ and Thomas F. Krauss^2,*^

^1^ State Key Laboratory of Optoelectronic Materials and Technologies, School of Physics and Engineering, Sun-Yat Sen University, Guangzhou, 510275, China

^2^ Department of Physics, University of York, York, YO10 5DD, UK

^3^ Raith Service & Support Team, Raith GmbH, 44263, German

^4^School of Engineering of São Carlos, University of São Paulo, Av. Trabalhador Sãocarlense, 400, São Carlos-SP, Brazil.

^*^Corresponding author: Thomas F. Krauss

*thomas.krauss@york.ac.uk*

^†^These authors contributed equally to this work and should be regarded as co-first authors.

**S1 – Spatial resolution effect and Fourier transform spectrum**

The Fourier transform spectra depend on the quality (magnification and resolution) of the relative real space, and is associated with the spatial resolution. In our previous work [23], we established that the resolution for a given nanostructure can be relaxed to 16 bit without impacting on the light trapping performance. Here, we demonstrate this effect by reducing the resolution of the same pattern (the unit cell in Figure S1a) from 128 bit to 4 bit, and assess the short-circuit current as a function of pattern resolution. The real space and the corresponding Fourier representation of different resolutions are shown in Figure S1 (b-g). The targeted Fourier region is highlighted by white circles. From Figure S1 (b-e), we can see that the resolution of the image decreases from 128 bit to 16 bit, while the corresponding Fourier transform spectrum still satisfies the target region. Figure S1(f-g) shows how the resolution of the unit cell decreases below the threshold, and the Fourier spectra are losing components in the highlighted region, which result in the loss of the excitation of quasi-guided mode for coupling [23] and the overall absorption drops. This trend is clearly shown in Figure S1(h). Therefore, the Fourier transform spectrum depends on the resolution of real space image and the absorption is more related to the Fourier transform spectrum.


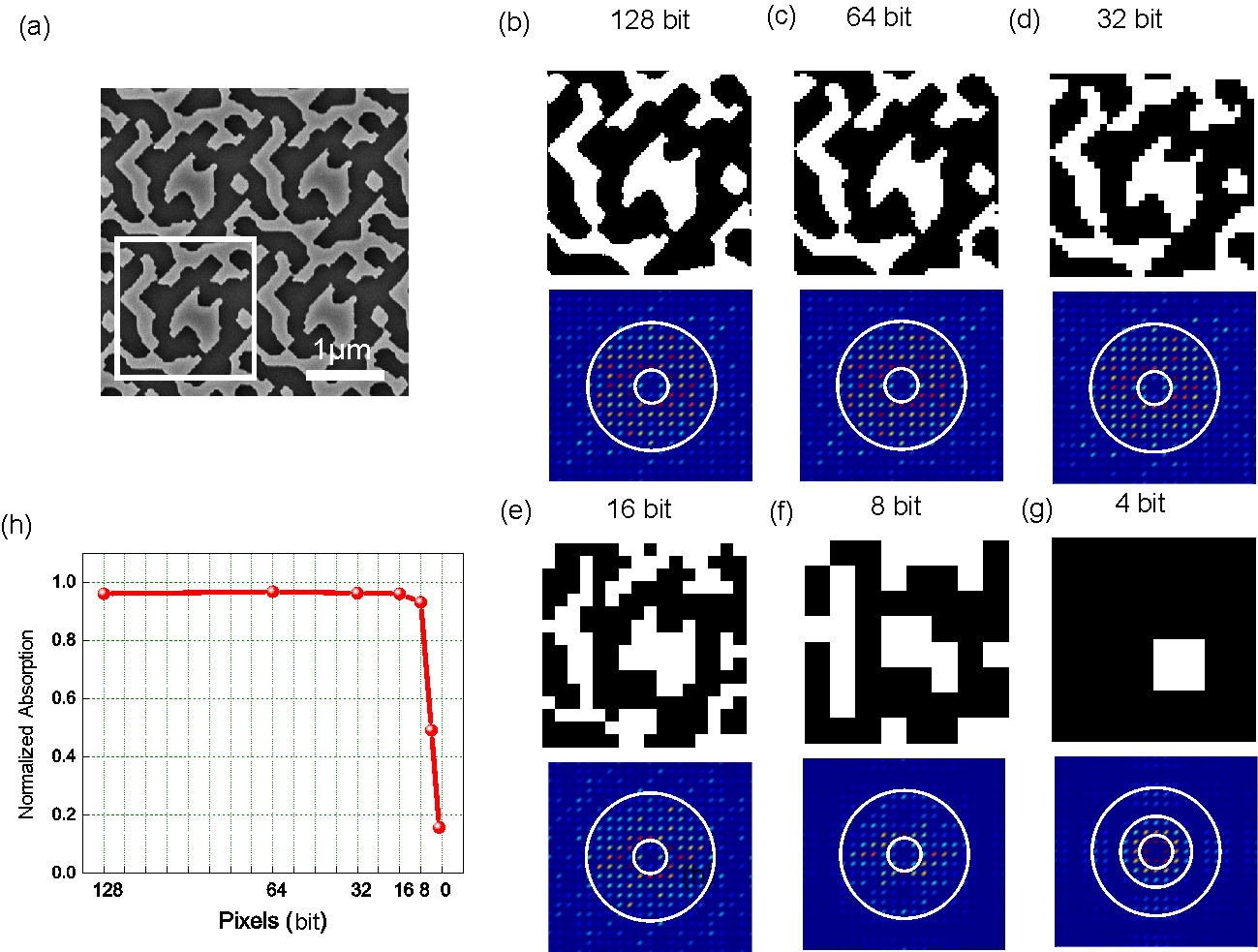


Figure S1 (a) SEM image of the quasi-random supercell, the highlighted rectangle region indicating the unit cell. (b-g) Real space and corresponding Fourier distribution of the unit cell shown in (a). The resolution of the real space image is reduced from 128 bit to 4 bit. (h) Integrated absorption as a function of spatial resolution.

**S2 – The choice of dose**

From figure 4 in the manuscript, we plot the trend of dose as a function of development time. Here, we illustrate how to identify the dose that could both reduce the writing time and keep the high photonic performance. As an example, we use the data that are shown in Figure 4 (blue curve, developing for 20 minutes at 22℃). First, we calculate the short-circuit current for each dose, then plot the short-circuit current as a function of the dose and the corresponding writing time (Figure S2). Finally, choose the “appropriate” dose which require less writing time but also has the highest short-circuit current. Figure S2 shows that by reducing dose from 108 to 58.5 μC/cm^2^, we could obtain the maximum short-circuit current while still keeping high speed writing.


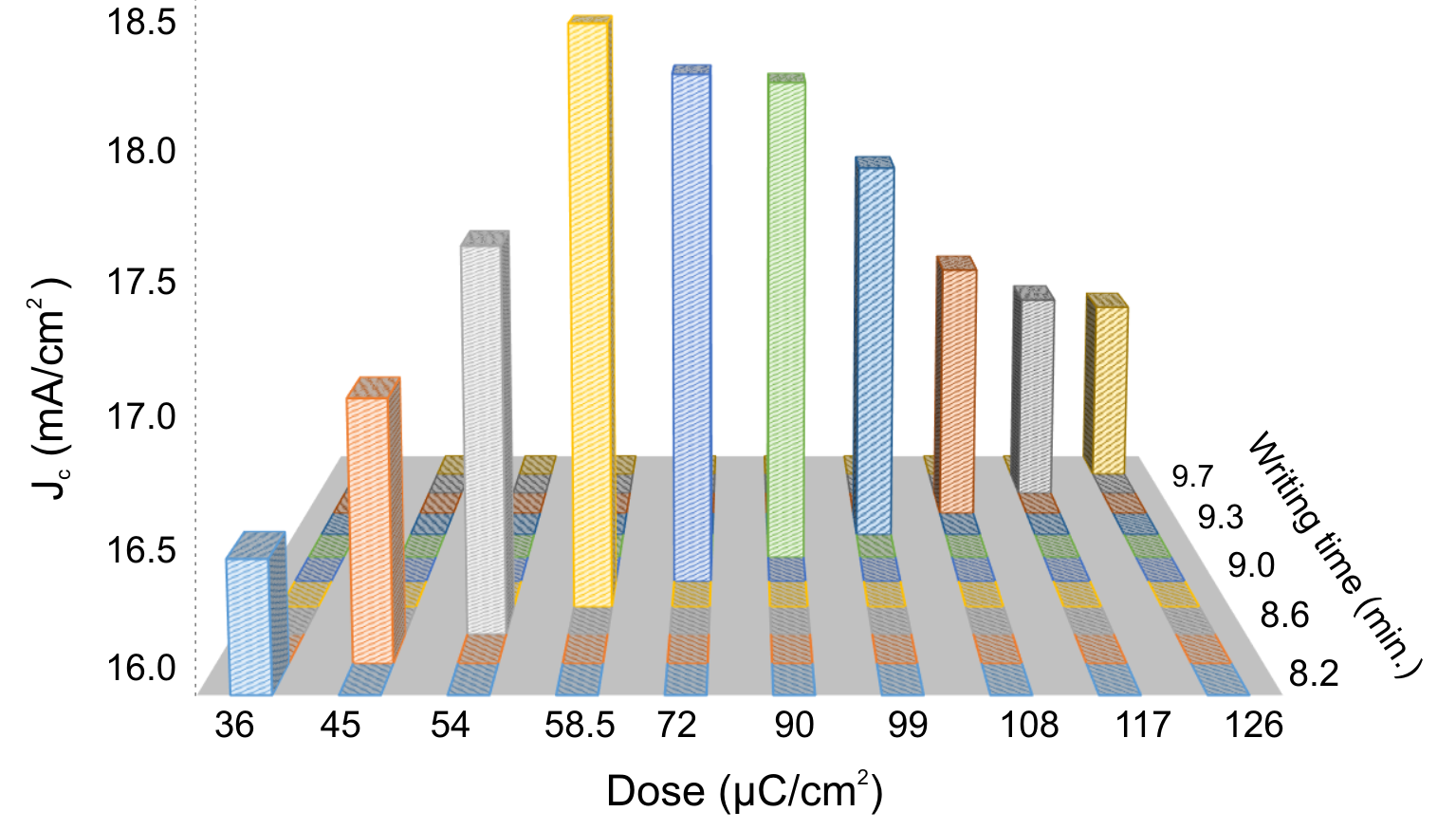


Figure S3 The relationship of dose, writing time, and short-circuit current.
